# The impact of the COVID-19 pandemic on neurofibromatosis clinical care and research

**DOI:** 10.1186/s13023-021-01711-w

**Published:** 2021-02-01

**Authors:** Heather B. Radtke, Bonita P. Klein-Tasman, Vanessa L. Merker, Pamela Knight, Nicole J. Ullrich, Justin T. Jordan, Bruce Korf, Scott R. Plotkin

**Affiliations:** 1 Children’s Tumor Foundation, New York, NY USA; 2 Medical College of Wisconsin, Milwaukee, WI USA; 3 University of WI – Milwaukee, Milwaukee, WI USA; 4 Edith Nourse Rogers Memorial Veterans Hospital, Bedford, MA USA; 5 Boston Children’s Hospital, Boston, MA USA; 6 Dana-Farber Cancer Institute, Boston, MA USA; 7 Massachusetts General Hospital, Boston, MA USA; 8 University of Alabama At Birmingham, Birmingham, AL USA

**Keywords:** COVID-19, Clinical care, Research, Rare disease, Neurofibromatosis, Telehealth, Telemedicine

## Abstract

**Purpose:**

The coronavirus disease 2019 (COVID-19) pandemic has had unprecedented impact on the provision of medical care for genetic disorders. The purpose of this study was to assess the effects of the pandemic on neurofibromatosis (NF) care and research.

**Methods:**

Sixty-three United States NF clinics were surveyed to identify the impact of the pandemic on clinician role, patient volume, continuity of guideline-driven surveillance, research protocols, and use of (and satisfaction with) telehealth for the delivery of NF care.

**Results:**

Fifty-two clinic directors or their representatives completed the survey (83% response rate). About 2/3 of the clinics reported a greater than 50% decrease in the number of available patient appointments, and modified clinical surveillance and research protocols. Fifty-one clinics (98%) newly instituted telehealth during the pandemic. Barriers to telehealth prior to the pandemic were insurance reimbursement concerns and lack of infrastructure. Since telehealth was initiated, high provider satisfaction was reported with ease of use. The most common area of concern was related to inability to perform a physical examination.

**Conclusion:**

Results show marked impacts on NF care and research since the beginning of the pandemic, with potential long-term changes related to the introduction (or adoption) of telehealth for clinical care.

## Introduction

### Neurofibromatoses

Neurofibromatosis type 1 (NF1), neurofibromatosis type 2 (NF2), and schwannomatosis (SWN) are a group of genetic tumor predisposition syndromes, collectively called the neurofibromatoses (NF). All types of NF predispose to nerve sheath tumors (i.e., neurofibromas or schwannomas) throughout the body and affect up to 1 in 3,000 individuals. There is significant variability in the timing, severity, and presence of specific clinical manifestations in individuals with NF. Each type of NF is progressive and requires lifelong medical monitoring and care [1]. Although there is no cure and there are limited treatments for NF, selumetinib (Koselugo) received FDA approval in April 2020 for the treatment of symptomatic, inoperable plexiform neurofibromas in pediatric NF1 patients [2]. There is an urgent need to identify new treatments for NF1, NF2, and SWN, for which clinical trials are highly concentrated in specialty clinics [3].

### NF clinic network

The NF Clinic Network (NFCN) is a group of member NF clinics in the US that have been approved by the Clinical Care Advisory Board of the Children’s Tumor Foundation (CTF) based on NF expertise, patient volume, multidisciplinary approach, and research support [4]. There are currently 63 NFCN clinics located in 32 states [5].

### COVID-19 pandemic

The coronavirus disease 2019 (COVID-19) first appeared in late 2019 and spread quickly worldwide [6]. During the initial wave of the U.S. pandemic in March 2020, many states initiated stay-at-home orders and health safety guidelines [7]. These regulations had a drastic effect on all aspects of life, including both access to and provision of medical care. As resources were diverted to care for COVID-19 patients, routine clinical care and elective procedures were temporarily paused in many locations. While such measures were important to address the pandemic, there were likely negative collateral effects on the management of chronic conditions [8]. To date, the impact of COVID-19 on clinical care and research for genetic disorders has largely been unexamined. The goal of this survey was to investigate the impact of COVID-19 on NF care delivery and research.

## Materials and methods

NFCN member clinics were invited to participate in an online survey administered by SurveyMonkey. (Additional file 1) NFCN clinic directors come from a variety of disciplines including genetics (40%), neurology (26%), neuro-oncology (17%), hematology/oncology (7%) and other subspecialties. The majority of clinics provide both pediatric and adult NF care. Over one-half of clinics report that they have on-site clinical trials or NF research and 18/63 clinics are part of the NF Clinical Trials Consortium [9]. Together, the NF clinics provide care to over 15,000 individuals with NF per year [5].

**Fig. 1:**
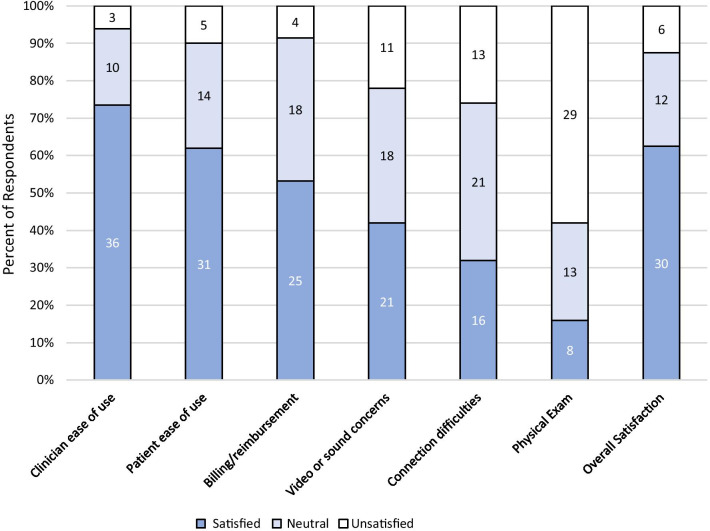
Clinician Satisfaction with Telehealth for NF Patient Care. Over half of clinics reported overall positive satisfaction with telehealth for NF patient care with areas of least satisfaction with ability to perform physical examination and technical difficulties

One response per clinic was requested from each clinic. Invitations were emailed on May 7, 2020 with one follow up email to non-responders on May 22, 2020. The survey was closed on June 2, 2020. Respondents were asked a series of questions related to current clinical care and research practices, as well as estimated relative clinic volume between April 1–30, 2020, to allow consistent comparison between clinics. Clinician satisfaction with NF care provision by telehealth was assessed using a Likert rating scale (1: very satisfied to 5: not at all satisfied) (Fig. 1). Respondents were offered a $15 gift card for completion of the survey, which took an average of 10 min to complete.

## Results

### Participants

Sixty surveys were completed. Eight responses were excluded from analysis (N = 4 duplicate response from same clinic; N = 4 incomplete survey response). Response rate was 83% (52/63 clinics) and respondents included 47 clinic directors and 5 clinic coordinators. Main clinic focus was identified as pediatric (36%), adult (10%) or a combination of pediatric and adult (54%), with representation from all over the US.

### Clinic director staffing availability

Two clinicians (one geneticist and one neurologist) were required to be redeployed to another service and/or on the frontline to assist with COVID-19 patients and two (neurologists) volunteered to do so. Six (12%) of the directors were partially furloughed with reduction in clinical hours.

### Clinic volume

Clinics were asked to provide an estimate of patient volume during April 2020, compared to pre-COVID average volume (including both in-person and telehealth visits) (Table 1). The typical number of NF patients seen in each clinic before the pandemic ranged widely from 3 to 35 patients per week. Thirty-four clinics (65%) reported reduced clinic volume by ≥ 50%; within these, 18 clinics (35%) with volume reduction by ≥ 75%, and 3 clinics (6%) with no reported clinic volume (100% reduction). Two clinics reported > 100% of their pre-COVID clinic volume.

**Table 1:** Impact of COVID-19 on NF clinic patient volume

Estimated patient volume in April 2020 compared to pre-COVID-19 pandemic (n = 52)	
0%	3 (5.8%)
1–25%	15 (28.9%)
26–50%	16 (30.8%)
51–75%	7 (13.5%)
76–100%	9 (17.3%)
> 100%	2 (3.9%)

Estimated percentage of clinic patient volume delivered by telehealth (n = 49*)	
100%	5 (10.2%)
75–99%	32 (65.3%)
50–74%	5 (10.2%)
25–49%	5 (10.2%)
0–24%	2 (4.0%)

Based on respondent estimates, a vast majority of clinics reported a decrease in patient volume in April 2020 compared to prior to the pandemic, and most clinics were using telehealth for a majority of their NF clinic patients

*Excludes three clinics reporting no patient visits in April 2020

### Triaging of urgent issues

Most clinics continued to see urgent NF patients (92%), and most clinics (79%) reported that they were conducting urgent visits via telehealth. All clinics reported that patients were able to have urgent MRIs or testing done at the onsite medical center or an affiliate site; 41% reported that patients could similarly have routine non-urgent MRIs or testing. However, 43% of directors reported non-urgent MRIs and testing were being electively deferred and 16% indicated that non-urgent testing could not be performed.

### Impact on access to novel therapies

Selumetinib (Koselugo) for the treatment of plexiform neurofibromas in pediatric NF1 patients received FDA approval during the COVID-19 pandemic. While 22% of NF clinics had already treated or discussed treatment with eligible patients, 63% were waiting to discuss treatment until patients’ next appointments (which as noted, had been delayed due to COVID-19 for many patients) and 12% were deferring starting new patients on treatment until after resolution of the pandemic.

### Clinical trials participation

Respondents were asked about their institution’s current policy regarding NF clinical trials and could select more than one response given that clinics may have more than one clinical trial at their institution. Although 39% of clinics were able to continue recommended treatment and surveillance protocols, in over half of currently enrolled patients, protocols were either modified (37%) or temporarily deferred (14%). In addition, 43% of clinics delayed enrolling patients into existing trials and 29% delayed IRB review/activation of new clinical trials.

### Impact of pandemic on provision of telehealth

Only one of the clinics surveyed was using telehealth for follow-up patient visits prior to COVID-19. The most common reported barriers reported for not using telehealth prior to the pandemic were: insurance/reimbursement concerns (63%), lack of institutional setup for telehealth services (59%), and safety concerns, which included limitations of physical exam using telehealth (49%). Additional concerns raised included technological issues or difficulties using a telehealth system (37%), legal concerns (e.g., medical licensure issues; 35%), security/privacy concerns (24%), lack of interest in using telehealth by leadership and/or colleagues (24%), and no identified need for telehealth (24%). Within the open-ended narrative response, some concerns regarding access issues were raised including challenges patients may have accessing video televisits and challenges with integrating interpreter services to address hearing difficulties, which are common with NF2.

Since the COVID-19 pandemic, 51/52 (98%) of clinics were using telehealth for NF patients; the remaining clinic planned to begin telehealth in the next three months. A majority of clinics reported seeing at least half of patients by telehealth at the survey endpoint, less than 3 months from the beginning of the pandemic (Table 1). New, follow-up, and genetic counseling appointments included full evaluations (84%), problem-focused discussions of medical issues (80%), follow up of previous recommendations (73%), discussion of results (65%), and problem-focused discussions of neurocognitive/psychosocial issues (51%). Clinics used a wide variety of telehealth platforms, some of which were integrated within the electronic medical record systems. The most frequently used platforms used were Zoom (58%) and Doximity (23%).

As indicated above and in Table 1, most clinics (86%) reported that the majority of their total clinic volume was currently seen via telehealth. Use of video conferencing was more common than use of telephone alone for appointments.

### Clinician satisfaction with telehealth

Clinic satisfaction with telehealth is indicated in Fig. 1. Overall, 63% of respondents were satisfied or very satisfied with the use of telehealth services for NF patient care. The ease of use of telehealth by clinicians was the highest area of satisfaction, with patient ease of use and billing reimbursement rated as neutral to positive. Technical issues and connection difficulties were rated in the neutral to negative range. The lowest satisfaction was with the ability to perform a physical examination, with 29 clinicians (72%) indicating that they were not satisfied or not at all satisfied with this aspect of telehealth.

### Plan for continued use of telehealth for NF patient care

A majority (84%) of clinics indicated they would continue to use telehealth for NF patient care, provided maintenance of adequate insurance coverage. The remaining clinics said they were unsure whether they will continue telehealth. Within the open-ended narrative response, some clinicians reported that telehealth would be used only in certain cases, such as for stable or uncomplicated follow-up patients, families traveling a distance or reluctant to come to the hospital, triaging of patient needs; telehealth visits in those circumstances would be alternated with in-person visits.

## Discussion

This study, conducted fairly early in the course of the pandemic, elucidates the impact of the COVID-19 pandemic on clinical care for a group of rare, neurogenetic disorders [the neurofibromatoses (NF)]. Immediate impact includes changes to clinician roles, patient volume, and medical treatment/surveillance protocols that reduced the availability of routine NF-specific care. Delays in appointments or testing, even in routine situations, may postpone recognition of NF-related complications. The pandemic prompted a pivot from face-to-face medical care to telehealth, in order to meet healthcare needs. Clinical trials participation was also impacted, with half of clinics reporting either modification or deferral of existing clinical trials protocols and delays or suspensions with new research protocols. Given the lack of approved treatment options for most manifestations of NF, even temporary delays in clinical trial research may be devastating to many NF families and may slow NF research directed towards finding new treatments. Further research is needed about the specific considerations for use of telehealth approaches within clinical trials research.

Despite these challenges, telehealth was rapidly adopted by NF specialty clinics within 2–3 months, going from 2% of clinics pre-pandemic to 98% of clinics during the pandemic. A majority of clinics reported that this will likely continue for at least some patients if insurance reimbursement for these services continues. Further exploration of barriers to telehealth and clinician/patient satisfaction ratings of telehealth will guide future provision of remote care for rare, genetic disorders. Remote care options may be especially beneficial for individuals with rare conditions and their families given that clinicians expert in their care may not be readily accessible locally [4].

Clinics reported a wide range of pre-pandemic barriers to adoption of telehealth, some of which were at least temporarily mitigated by COVID-19 related policy changes, such as suspension of HIPAA rules regarding the security of telehealth platforms, licensure exceptions for the provision of care across some state lines, expanded reimbursement for telehealth and increased institutional support for telehealth. New barriers have emerged due to the rapid changes, including stress to IT systems, technological issues with set-up, limited IT support capacity and inability to supplement telehealth with in-person physical exams due to travel restrictions.

Our results suggest that to sustain use of telehealth for genetic diseases, technical support and training for both clinicians and patients need to improve, in addition to permanent changes to state licensure requirements and insurance reimbursement. Telehealth methods were new to the operations of these clinics at the time of survey completion, and institutional support was also new; it is possible that satisfaction would be different in the time period since the survey, with more personal and institutional experience. As the use of telehealth continues, care must be given to ensure the protection of personal data. In addition, the identification of specific patient factors, such as socioeconomic status or other barriers that may prevent equal access to care should be investigated further. Finally, a survey of patient experiences with telehealth is currently underway [10] and additional studies assessing patient satisfaction are critical to evaluating and optimizing telehealth utility and experiences of both clinicians and patients.

## Supplementary information

**Additional file 1**. Survey Instrument.

## Data Availability

The datasets used and/or analyzed during the current study are available from the corresponding author on reasonable request.
