# Supplementary material for: The impact of the COVID-19 pandemic on neurofibromatosis clinical care and research
Source: Orphanet J Rare Dis. 2021 Feb 1;16:61. doi: 10.1186/s13023-021-01711-w (PMC7848872; doi:10.1186/s13023-021-01711-w)
Supplement: Supplementary file 1 — Additional file 1. Survey Instrument. [file 13023_2021_1711_MOESM1_ESM.pdf]

Supplement 1: Survey Questions

The Impact of the COVID-19 Pandemic on Neurofibromatosis Care and Research

Although any NF clinic staff may complete the survey, please note that we request information about and perspectives of the NF clinic director(s).

* 1. Please provide your information.

Name (individual completing the survey) [ ]

Clinic Role [ ]

Email [ ]

Clinic Director(s) [ ]

Hospital/Institution [ ]

State [ ]

* 2. Please indicate the main focus of your NF clinic.

- ○ Pediatric
- ○ Adult
- ○ Pediatric and Adult

Comment [ ]

* 3. Has your clinic role changed at all during the COVID-19 pandemic? (select all that apply)

- [ ] Required to redeploy to another service and/or be on the front line to assist with COVID-19 patients
- [ ] Volunteered to redeploy to another service and/or be on the front line to assist with COVID-19 patients
- [ ] Worked remotely from home
- [ ] Worked a combination of onsite and remote
- [ ] Hours reduced and/or partial furlough
- [ ] Complete furlough
- [ ] Role remained unchanged

Comment [ ]

* 4. Approximately how many NF outpatients per week did your clinic see prior to the COVID-19 pandemic?
This includes NF1, NF2 and schwannomatosis patients.

* 5. Please indicate your best estimate of patient outpatient volume from April 1 – April 30 compared to prior to the start of the COVID-19 pandemic. (including both in-person and telehealth visits)

- 0% of pre-COVID-19 patient volume
- 1-25% of pre-COVID-19 patient volume
- 26-50% of pre-COVID-19 patient volume
- 51-75% of pre-COVID-19 patient volume
- 76-100% of pre-COVID-19 patient volume
- >100% of pre-COVID-19 patient volume

Comment

* 6. Is your clinic currently seeing ***urgent*** NF patients, in person (in an outpatient setting)?
The term "urgent" applies to a patient having an acute, possibly NF-related issue, with a potential to cause long-term damage or life-threatening complications. It might also apply to highly anxious or concerned patients.

- Yes
- No

Comment

* 7. Is your clinic currently seeing ***non-urgent*** NF patients, in person in an outpatient setting?

- Yes
- No

Comment

* 8. What percent of your total clinic patient volume is currently being seen <u>in person</u> (in an outpatient setting)?

- ◯ 0%
- ◯ 1-25%
- ◯ 26-50%
- ◯ 51-75%
- ◯ 76-100%

Comment

* 9. Is your clinic currently seeing ***urgent*** NF patients, by <u>telehealth</u>?

Telehealth applies to both video and/or telephone visits.

- ◯ Yes
- ◯ No

Comment

* 10. Is your clinic currently seeing ***non-urgent*** NF patients, by <u>telehealth</u>?

- ◯ Yes
- ◯ No

Comment

* 11. What percent of your total current NF patient volume is being “seen” by <u>telephone</u>?

- ◯ 0%
- ◯ 1-25%
- ◯ 26-50%
- ◯ 51-75%
- ◯ 76-100%

Comment

* 12. What percent of your total current NF patient volume is being “seen” by video?

- ○ 0%
- ○ 1-25%
- ○ 26-50%
- ○ 51-75%
- ○ 76-100%

Comment

[ ]

* 13. Prior to the COVID-19 pandemic, was your clinic using telehealth for NF patients?

- ○ Yes
- ○ No

Comment

[ ]

* 14. Please indicate the reasons or barriers for not using telehealth for NF patients in the past: (select all that apply)

- [ ] No identified patient need for telehealth
- [ ] Don’t think patients will like telehealth
- [ ] Concerns telehealth will decrease productivity/slow things down
- [ ] Safety concerns (including limitations of physical exam using telehealth)
- [ ] Lack of time to initiate telehealth/difficulty learning telehealth system
- [ ] Technological issues/difficulty using telehealth system
- [ ] Clinical leadership and/or colleagues not interested in using telehealth
- [ ] Institution/department did not have set up for telehealth services
- [ ] Telehealth not compatible with other technologies (like electronic medical record system)
- [ ] Inadequate IT support for telehealth
- [ ] Insurance/reimbursement concerns
- [ ] Legal concerns (ex. medical licensure issues)
- [ ] Security/privacy concerns

Comment

[ ]

* 15. What type of NF patient visits was your clinic providing via telehealth prior to the COVID-19 pandemic? (select all that apply)

- [ ] New patient appointments
- [ ] Follow up patient appointments
- [ ] Genetic counseling sessions

Comment

* 16. What type of NF patient evaluations was your clinic providing via telehealth prior to the COVID-19 pandemic? (select all that apply)

- [ ] Full evaluation within the capabilities of telehealth services
- [ ] Problem focused discussion (medical issues)
- [ ] Problem focused discussion (neurocognitive/psychosocial issues)
- [ ] Check-in to review status of previous recommendations
- [ ] Results disclosure

Comment

* 17. Is your clinic currently providing via telehealth services for NF patients?

- ( ) Yes
- ( ) No, and clinic has no plans to do so within the next 3 months
- ( ) No, but plan to do so within the next 3 months

Comment

* 18. What type of NF patient visits is your clinic currently providing via telehealth? (select all that apply)

- [ ] New patient appointments
- [ ] Follow up patient appointments
- [ ] Genetic counseling sessions

Comment

* 19. What type of NF patient evaluations is your clinic currently providing via telehealth? (select all that apply)

- [ ] Full evaluation within the capabilities of telehealth services
- [ ] Problem focused discussion (medical issues)
- [ ] Problem focused discussion (neurocognitive/psychosocial issues)
- [ ] Check-in to review status of previous recommendations
- [ ] Results disclosure

Comment

20. What platform(s) and/or software does your clinic use for your NF telehealth service? (select all that apply)

- [ ] Zoom
- [ ] FaceTime
- [ ] Google Hangouts/Meet
- [ ] Microsoft Teams
- [ ] Skype
- [ ] Polycom
- [ ] American Well
- [ ] Doxy.me
- [ ] RingCentral
- [ ] Doximity
- [ ] Not sure

Other (please specify)

21. Is your telehealth platform linked to your institution's electronic medical record system?

- ( ) Yes
- ( ) No
- ( ) Not sure

Comment

22. Regarding your clinic's telehealth experience with NF patients, please indicate your clinic's satisfaction with the following:

	Very satisfied				Not at all satisfied
Ease of use (for clinician)	◯	◯	◯	◯	◯
Ease of use (for patients)	◯	◯	◯	◯	◯
Connection difficulties (problems logging in or interruption in connection)	◯	◯	◯	◯	◯
Technical concerns (video or sound issues)	◯	◯	◯	◯	◯
Billing/reimbursement	◯	◯	◯	◯	◯
Ability to perform a physical examination	◯	◯	◯	◯	◯
**Please rate your overall satisfaction with telehealth at your institution**	◯	◯	◯	◯	◯

Comment

23. If payment for telehealth services were covered by insurance, would your clinic continue using it for NF patients once the current crisis has ended?

- ◯ Yes
- ◯ No
- ◯ Not sure

Comment

* 24. Please indicate the reasons or barriers for not planning to use telehealth for NF patients in the near future: (select all that apply)

- [ ] Lack of resources at your home (such as appropriate hardware, private space for video visits, etc.)
- [ ] No identified patient need for telehealth
- [ ] Don't think patients will like telehealth
- [ ] Concerns telehealth will decrease productivity/slow things down
- [ ] Safety concerns (including limitations of physical exam using telehealth)
- [ ] Lack of time to initiate telehealth/difficulty learning telehealth system
- [ ] Technological issues/difficulty using telehealth system
- [ ] Clinical leadership and/or colleagues not interested in using telehealth
- [ ] Institution/department did not have set up for telehealth services
- [ ] Telehealth not compatible with other technologies (like electronic medical record system)
- [ ] Inadequate IT support for telehealth
- [ ] Insurance/reimbursement concerns
- [ ] Legal concerns (ex. medical licensure issues)
- [ ] Security/privacy concerns

Comment

* 25. If a patient needs an ***urgent*** MRI or other test during this COVID-19 crisis, are they able to have it performed at your facility?

- ○ Yes – at onsite medical center
- ○ Yes - at an affiliated site
- ○ No

Comment

* 26. If a patient needs a ***non-urgent*** (routine) MRI or other test during this COVID-19 crisis, are they able to have it performed at your facility?

- ◯ Yes - at onsite medical center
- ◯ Yes – at an affiliated site
- ◯ Yes, but often being deferred
- ◯ No

Comment

* 27. For any clinical trials open to NF patients at your institution, which of the following describes your institution's current policy: (select all that apply)

- ☐ Continued treatment and surveillance of patients per recommended protocol
- ☐ Continued treatment and surveillance but with a modified protocol
- ☐ Temporarily deferred treatment and surveillance of currently enrolled patients
- ☐ Delayed enrolling new patients onto an existing protocol
- ☐ Delayed IRB review/activation of a new clinical trial

Comment

* 28. If any of your patients are currently receiving a non-FDA approved or off-label medication for an NF indication, have they been able to continue with treatment and surveillance? (select all that apply)

- ☐ Continued treatment and surveillance of patients per recommended protocol
- ☐ Continued treatment and surveillance but with a modified protocol
- ☐ Temporarily deferred treatment and surveillance

Comment

* 29. With the recent FDA approval of selumetinib (Koselugo), when will eligible patients in your clinic be able to start this treatment (pending insurance approval)? (select all that apply)

- [ ] All eligible patients were already on treatment through Expanded Access program
- [ ] All eligible patients were already on treatment through on-site clinical trial
- [ ] Once FDA approval was confirmed, eligible patients were contacted
- [ ] Once a patient is seen in clinic or by telehealth, will discuss the option of Koselugo if indicated
- [ ] Clinic has deferred starting any patients on new treatment until after pandemic resolves

Comment

30. Feel free to provide additional comments on the impact of the pandemic on you, your NF practice, or your NF patients.
